# Staple-line leak post primary sleeve gastrectomy. A two patient case series and literature review

**DOI:** 10.1016/j.amsu.2019.06.014

**Published:** 2019-07-02

**Authors:** Guo Hou Loo, Reynu Rajan, Nik Ritza Kosai Nik Mahmood

**Affiliations:** a Surgical Trainee, Department of General Surgery, Faculty of Medicine, The National University of Malaysia, Jalan Yaacob Latiff, Bandar Tun Razak, Postcode 56000, Selangor, Malaysia; b Consultant Bariatric Surgeon, Department of Surgery, Faculty of Medicine, The National University of Malaysia, Jalan Yaacob Latiff, Bandar Tun Razak, Postcode 56000, Selangor, Malaysia; c Consultant Upper Gastrointestinal & Bariatric Surgeon, Head of Unit of Upper Gastrointestinal and Minimally Invasive Surgery, Department of Surgery, Faculty of Medicine, The National University of Malaysia, Jalan Yaacob Latiff, Bandar Tun Razak, Postcode 56000, Selangor, Malaysia

**Keywords:** Bariatric surgery, Complications, Southeast Asia, Asian population, Endoscopic stenting

## Abstract

There is an increasing trend in the number of bariatric surgeries performed worldwide, partly because bariatric surgery is the most effective treatment for morbid obesity. Sleeve gastrectomy (SG) remains the most common bariatric surgery procedure performed, representing more than 50% of all primary bariatric interventions. Major surgical complications of SG include staple-line bleeding, leaking, and stenosis. A leak along the staple-line most commonly occurs at the gastroesophageal junction (GOJ).

From January 2018 to December 2018, our centre performed 226 bariatric procedures, of which, 97.8% were primary bariatric procedures. The mean age and BMI were 38.7±8.3 years and 44 kg/m2, respectively. Out of the 202 primary SG performed, we encountered two cases of a staple-line leak (0.99%). This is the first reported case series of SG leaks from the Southeast Asia region. A summary of their characteristics, clinical presentation, subsequent management, and the outcome is discussed.

Based on the latest available evidence from the literature, several methods may decrease staple-line leaks in SG. These include the use of a bougie size greater than 40 Fr, routine use of methylene blue test during surgery, beginning transection at 2–6 cm from the pylorus, mobilising the fundus before transection, and staying away from the GOJ at the last firing. Other methods include the proper alignment of the staple-line, control of staple-line bleeding, and performing staple-line reinforcement. The management of a staple-line leak remains challenging due to limited systematic, evidence-based literature being available. Therefore, a tailored approach is needed to manage this complication.

## Highlights

• Major surgical complications of sleeve gastrectomy include staple-line bleeding, leaking, and stenosis.
• The incidence of leak after sleeve gastrectomy is reported to be 0.74%.
• We report a leak rate of 0.99%, based on 202 sleeve gastrectomy performed in a single centre.
• This is the first reported case series of SG leaks from the Southeast Asia region to the best of our knowledge.
• Based on the latest available evidence from the literature, several methods may decrease staple-line leaks in SG.
• It is crucial that patients with this complication are managed by a multidisciplinary team.

## 1 Introduction

The number of bariatric surgeries performed worldwide is increasing, partly because bariatric surgery is the most effective treatment for morbid obesity [1]. According to the International Federation for the Surgery of Obesity and Metabolic Disorders (IFSO) worldwide survey, in 2016, a total of 685,874 bariatric/metabolic surgeries were performed worldwide [1]. Sleeve gastrectomy remains the most common bariatric surgery procedure performed, representing more than 50% of all primary bariatric interventions [1]. The clinical advantages of sleeve gastrectomy include shorter operative time, low risk of complications, good weight loss for up to five years of follow-up, similar comorbidity improvements as Roux-en-Y gastric bypass (RYGB), no re-routing of intestines so no bowel obstruction from internal herniation, reduced risk of malabsorption, absence of foreign material, and the ability to be converted into other bariatric procedures [1,3,9,10].

However, there can be major surgical complications of sleeve gastrectomy, including staple-line bleeding, leakage, and staple-line stenosis [2,3]. A gastric leak along the lengthy staple-line most commonly occurs at the upper staple-line near the gastroesophageal junction (GOJ) [4,5]. The incidence of leak after sleeve gastrectomy is reported to be 0.74%, based on the latest study in 2011 [11], and is the second most common cause of death after sleeve gastrectomy, with an overall reported mortality rate of 0.4% [2]. Long-term complications include the development of de novo gastroesophageal reflux disease (GERD), erosive esophagitis, and Barrett's oesophagus [[12], [13], [14]].

From January 1st, 2018 to December 31st, 2018, our centre performed 226 bariatric procedures, of which, 97.8% were primary bariatric procedures. The mean age and BMI were 38.7±8.3 years and 44 kg/m2, respectively. Primary laparoscopic sleeve gastrectomies (n = 202) accounted for 89.4% of all bariatric procedures performed, and of these 202, we encountered two cases of a staple-line leak (0.99%). In both of our patients, the leak originated from the staple-line near the GOJ. This case series has been reported in line with the PROCESS 2018 criteria [21].

## 2 Aim

Because sleeve gastrectomy is commonly performed at our bariatric unit, we aimed to evaluate the best option for managing gastric leaks and review the preventive methods that can be employed.

## 3 Materials and methods

Between January 1st, 2018 and December 31st, 2018, we performed 226 bariatric surgery procedures at our centre, 89.4% (n = 202) of which consisted of laparoscopic sleeve gastrectomy (LSG). Written informed consent was obtained from each patient. Data regarding demographic and anthropometric characteristics, operation discharge summaries, and leak management were extracted and analysed. Ethical approval has been exempted by our institution's ethics committee (The National University of Malaysia's Ethics Committee) as this publication is a retrospective case series, provided that patients have given their informed written consent for the publication of this case series. This study has been registered with Thai Clinical Trial Registry with the TCTR ID TCTR20190606009. Two non-consecutive patients experienced staple-line leak after sleeve gastrectomy during this period. A summary of their characteristics, clinical presentation, subsequent management, and the outcome is provided.

### 3.1 Surgical procedure

A 5-trocar technique was employed for primary LSG. An upper gastrointestinal consultant with more than five years’ experience performed these procedures. A 36 Fr sized bougie was inserted after anaesthetic induction. Standard LSG is performed with a multiple-firing endoscopic stapler device. A standard methylene blue leak test was performed in all LSG patients. No routine reinforcement of stapler line is performed. Patients were allowed clear fluids on post-operative day one and had fluids only diet for two weeks. They were discharged three days after surgery under normal circumstances.

## 4 Results

### 4.1 Case 1

**Fig. 1:**
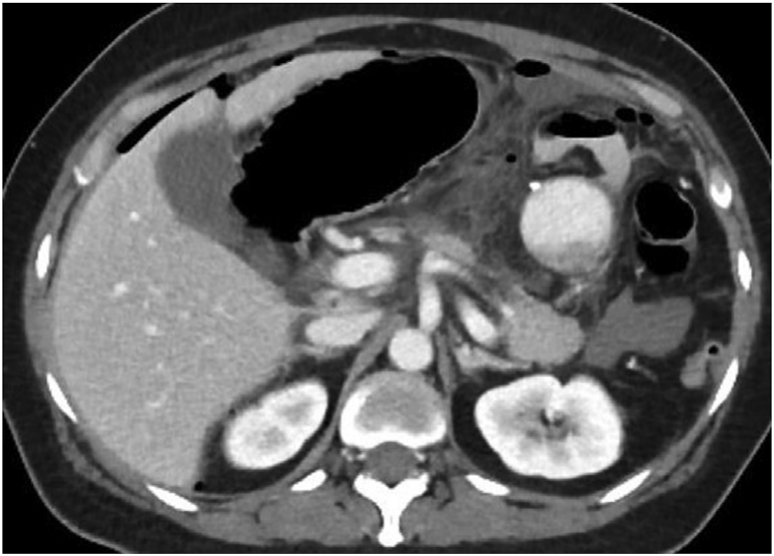
Case 1. Axial view of the CECT of the abdomen. The stomach is dilated with focal collection within the lesser sac, which contains oral contrast and air. This collection measures 3.8 × 6.9 × 11 cm in size and was suggestive of a staple-line leak.

**Fig. 2:**
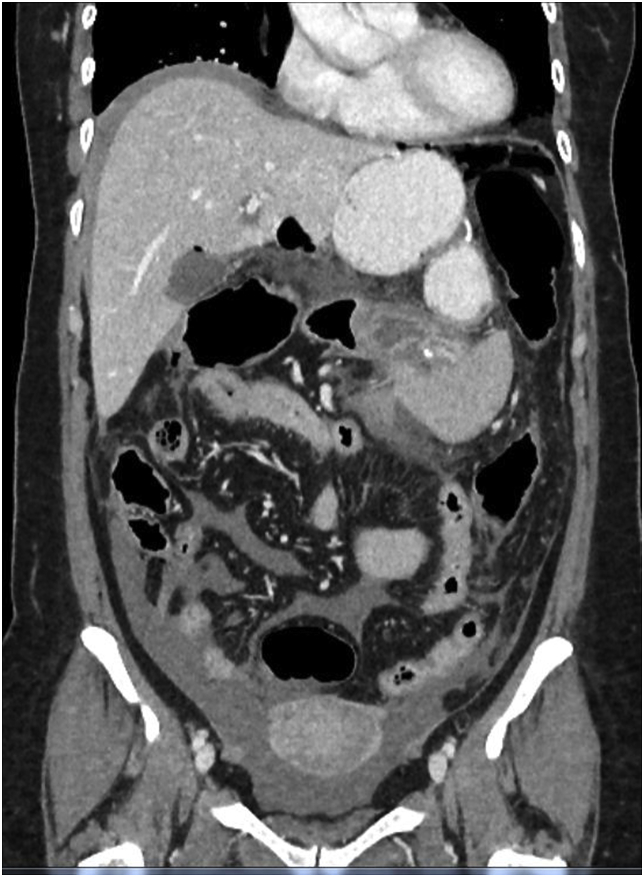
Case 1. Coronal view of the CECT of the abdomen and pelvis. Lesser sac collection is visualised, along with mesenteric fat stranding. Free fluids are seen throughout the abdomen and pelvis.

A 49-year-old lady with underlying non-alcoholic fatty liver disease (NAFLD) and GERD underwent LSG. She had a BMI of 35 kg/m2. Because of her lower BMI, the procedure was supposed to be a stand-alone procedure. On postoperative day three, she developed sudden onset abdominal pain, abdominal distension, and had a few episodes of passing loose stools. A leak was suspected, and an urgent contrast-enhanced computed tomography (CECT) of the abdomen and pelvis was performed. It revealed a collection within the lesser sac in keeping with a leak, possibly at the distal surgical site. There was no evidence of a stomach volvulus from the CT (Fig. 1, Fig. 2). The patient was resuscitated with fluids prior to surgery. She was then taken back to the operating room for laparoscopic exploration, and a small staple-line leak was noted adjacent to the GOJ. There were 2 L of seropurulent fluid in the peritoneal cavity, and there was a sleeved stomach volvulus causing a functional obstruction.

We performed a salvage RYGB on her, and a repeat CECT of her abdomen and pelvis on day four after surgery revealed the resolution of the previously seen lesser sac collection, with no evidence of extraluminal contrast. She had persistent tachycardia postoperatively and subsequently deteriorated clinically. This was likely due to severe intra-abdominal sepsis, which leads to multiorgan failure despite intensive care. Unfortunately, she passed away on postoperative day 20 (of the second operation) from septic shock, secondary to intra-abdominal sepsis.

### 4.2 Case 2

**Fig. 3:**
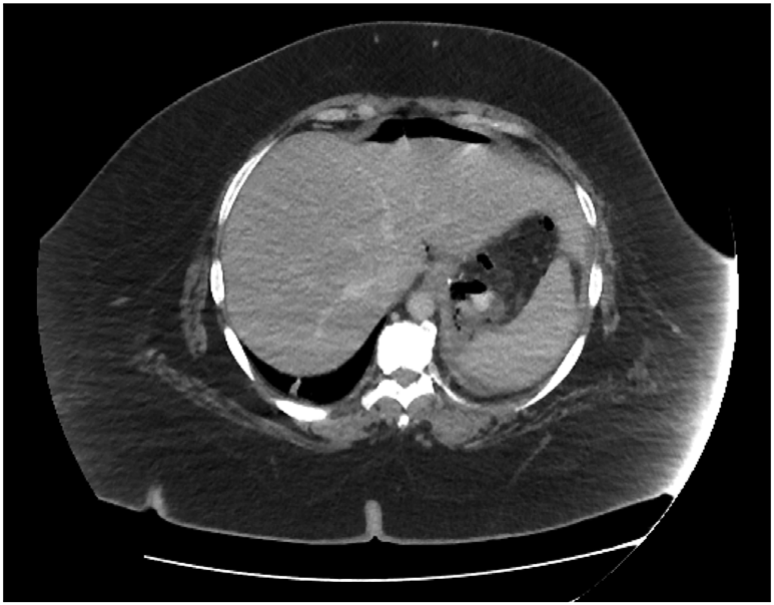
Case 2. Axial view of the CECT of the abdomen. Suspicious pooling of oral contrast with air locules are seen posterior to the stomach, suggestive of a leak.

**Fig. 4:**
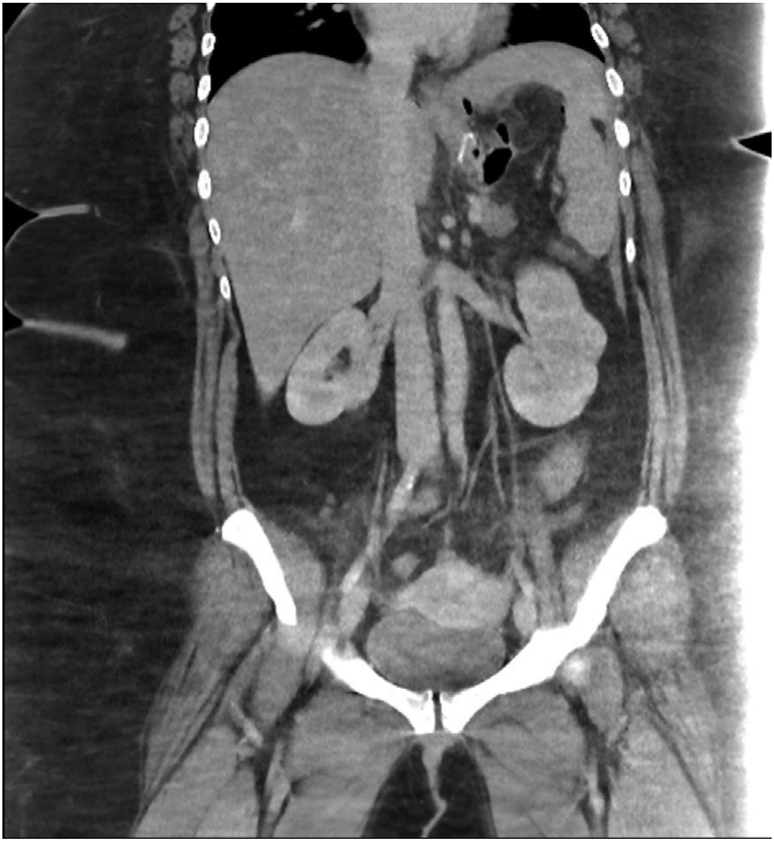
Case 2. Coronal view of the CECT of the abdomen and pelvis. A small amount of oral contrast is seen with surrounding free air.

A 39-year-old lady with a BMI of 74 kg/m^2^ underwent LSG. As she was in the super-super obese category, the procedure was supposed to be a staged procedure. She had underlying severe obstructive sleep apnoea (OSA), with an apnoea-hypopnoea index (AHI) of 45.5/hour, newly diagnosed type 2 diabetes mellitus, and essential hypertension. She did not require post-operative intensive care and was discharged well on postoperative day three. She was tolerating a liquid diet at home but came back on postoperative day 10 with complaints of left hypochondrium abdominal pain. An urgent CECT of the abdomen and pelvis revealed air pockets adjacent to the GOJ region with a small pooling of contrast, which was suspicious of a leak (Fig. 3, Fig. 4). She was started on broad-spectrum intravenous antibiotics and fluid resuscitated before definitive intervention was performed.

**Fig. 5:**
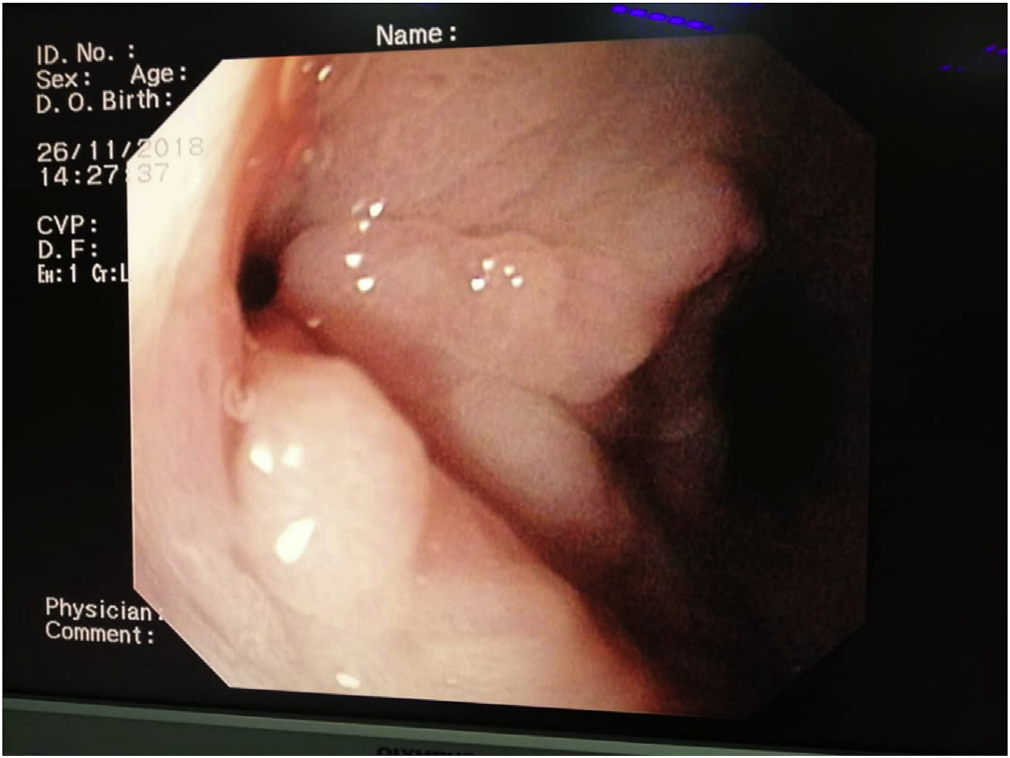
A repeat upper endoscopy five weeks after stent removal showed a walled-off perforation measuring 0.5 × 0.5 cm at the proximal gastric tube.

An urgent gastroscopy was performed and revealed a suspicious erythematous area just distal to the GOJ at the staple line. A 22 cm length oesophageal covered stent (Taewoong MEGA™) was deployed. Percutaneous drainage of the intra-abdominal collection was subsequently done. The stent was removed after five weeks, and a repeat gastroscopy revealed a walled-off perforation measuring 0.5 × 0.5 cm at the proximal gastric tube (Fig. 5). A 24 cm Gastro seal™ (M.I Tech) was placed. The stent was then removed after five weeks when the leaking site had healed. Patient was discharged well and during her last clinic follow up after six months, she has no abdominal symptoms and her BMI was 54 kgs/m^2^.

## 5 Discussion

Bariatric surgery is gaining popularity [1]. Surgery results in greater improvement in weight loss outcomes and weight-associated comorbidities when compared with non-surgical interventions, regardless of the type of procedure used [9]. The most severe complication of sleeve gastrectomy is a staple-line leak; most staple-line leaks are secondary to ischemic or mechanical issues, with the intraluminal pressure exceeding suture-line and tissue resistance [3]. Management options depend on the timing and clinical presentation of the leak [8,15,16]. Leaks are classified, based on observation periods, into acute, early, late, or chronic leaks [8]. An acute leak occurs when the time of presentation is within one week; an early leak, one to six weeks; a late leak, six weeks; and a chronic leak, after 12 weeks [8].

Out of 202 LSG cases over a year, two of our patients experienced a staple-line leak. Limitation of this case report includes the retrospective nature of this study, as well as a small sample size. It is, however, the first reported case series of LSG leaks from the Southeast Asia region to the best of our knowledge.

The first of our case suffered an acute leak, which was detected on postoperative day 3. As she was showing signs of systemic sepsis (persistent tachycardia and increasing abdominal pain), with radiological evidence of a staple-line leak, we elected to perform an immediate reoperation. An unstable patient with a contained or uncontained leak warrants an urgent reoperation [7]. Patients with fever and tachycardia but normal findings from upper endoscopy or other imaging studies require urgent reintervention or reoperation as well [7]. Performing an RYGB on this patient converted a high-pressure system to a lower pressure system, which is a treatment option for staple-line leaks [7].

In a stable patient with a proximal and mid-aspect gastric sleeve leak, the use of an endoscopic stent may be a viable option in an attempt to exclude the defect [7,15,20]. An upper endoscopy needs to be performed to assess the size and location of the leak, as well as the viability of the gastric sleeve [4]. As endoscopic covered stents decrease the intraluminal pressure, it allows a conducive environment for the leaking site to heal.

Our second patient presented to us on postoperative day 10. She had an early leak, and as she had no signs of systemic sepsis, we proceeded with endoscopic covered stent placement. The use of an endoscopic covered stent is a safe and effective option in the management of staple-line leaks after sleeve gastrectomy [4,7,15,20]. However, 30 days after the operation, the possibility of the leak site healing by exclusion using a stent is low [7]. Successful placement of a stent allows the patient to be fed orally, and if well, can be monitored as an outpatient [15]. However, the stent migration rate is high (58–59%), and they have to be removed after 6–8 weeks, as the gastric mucosa may be damaged if kept for a more extended period [15,22,23].

Other complications include tissue overgrowth (15%), ruptured stent cover (12%), food obstruction (6%), severe retrosternal pain (3.8%), oesophageal rupture during stent removal (3.8%), and haemorrhage (3.8%) [19]. Our second patient experienced severe retrosternal pain after the stent placement, which improved with proton pump inhibitor infusion. Some authors prefer to reserve stents for use in patients who do not recover with other methods of management such as laparoscopic washout and feeding jejunostomy [10], but ultimately, proper patient selection is essential in deciding whether an endoscopic stent is suitable, as it offers an alternative to surgery [4,19,20].

A more conservative method can be employed in a stable patient presenting with a late or chronic leak [7,16]. The International Sleeve Gastrectomy Expert Panel recommends at least 12 weeks after conservative therapy before reoperation to repair a proximal leak [7].

In our centre, primary sleeve gastrectomy is the most common bariatric procedure performed. Based on the latest available evidence from the literature, several methods used may decrease staple-line leaks in sleeve gastrectomy. The use of a bougie size >40 Fr may decrease staple-line leaks. This has been shown in a systematic review and meta-analysis by Parikh M. et al. (2013). They also showed that using a bougie size >40 Fr does not impact the percentage of estimated weight loss for up to 3 years [6]. However, an expert panel consensus by Rosenthal et al. (2012) advocated an optimal bougie size of 32–36 Fr [7]. They contended that using one < 32 Fr may increase complications, and using a bougie > 36 Fr could result in failure of weight loss [7]. At our centre, we routinely use a bougie size of 36 Fr.

At our centre, we routinely perform a methylene blue test on all of our LSG patients. This is because any staple-line leak detected intraoperatively may be repaired [16]. However, the International Sleeve Gastrectomy Expert Panel failed to reach a consensus about whether routine intraoperative leak tests should be performed [7]. The methylene blue test has been shown to have high sensitivity and specificity [16]. However, it should be remembered that a negative methylene blue test does not exclude a leak [16].

The surgical technique of beginning the gastric transection at 2–6 cm from the pylorus, mobilising the fundus completely before transection and staying away from the GOJ at the last firing is also crucial in preventing leaks. This recommendation is based on the expert panel consensus by Rosenthal et al. [7] However, in a systematic review by Parikh M. et al. (2013), no differences were found in the leak rate or weight loss between beginning transection within 5 cm from the pylorus and more than 5 cm away from the pylorus [6]. The recommendation to stay away from the GOJ is to avoid ischemic complications related to the transection of segmental vascularisation in this area [2]. We routinely begin our transection at 5 cm from the pylorus, completely mobilising the fundus prior to stomach transection, and the last stapler firing is performed 2 cm away from the GOJ.

Another crucial technical point to note is to avoid creating a spiral staple-line on the sleeved stomach by adequately aligning the staples. The proper orientation of the created sleeve is important and unequal traction of the stomach during stapler firing should be avoided [7]. If the anterior and the posterior wall of the stomach are not taken equally, there is a possibility of creating a spiral staple-line. The creation of a gastric tube that is not cylindrical results in high pressure, especially at the proximal part of the staple line [2]. A spiral gastric tube may lead to gastric volvulus, causing a functional obstruction, as a sleeved stomach is devoid of any fixation along the greater curvature, and the ensuing high-pressure system created may lead to a leak [2]. This is evident in our first patient, as she developed a staple-line leak from a gastric volvulus that was causing a functional obstruction.

Adequate haemostasis from the staple-line is vital. Haematoma at the staple-line may compromise tissue vitality and vascularity, predisposing a staple-line leak [7]. The International Sleeve Gastrectomy Expert Panel reached a consensus that staple-line reinforcement will lead to a decrease in bleeding from the staple line [7]. Various materials have been used to reinforce staple-lines; based on two systematic reviews, reinforcement using absorbable polymer membrane (APM) or bovine pericardial strips (BPS) are recommended [17,18]. Both systematic reviews agree that for reducing staple-line complications, staple-line reinforcement provides better results compared to no reinforcement [6,17,18], although it is at an increased cost [6]. The International Sleeve Gastrectomy Expert Panel agrees, however, that oversewing the staple-line is an acceptable option [7]. In our centre, we practice meticulous dissection and careful tissue handling to reduce bleeding. We do not reinforce our staple-line because of the potential increased cost incurred.

Sleeve gastrectomy (SG) remains the most common bariatric surgical procedure performed worldwide [1]. It is likely that this procedure will continue to be preferred in the future because of the above mentioned clinical advantages [1]. However, long term complications of SG, such as the development of de novo GERD, erosive esophagitis, and Barrett's oesophagus should be kept in mind [[12], [13], [14]]. The prevalence of Barrett's oesophagus five years after SG is 18.8%, and is associated with weight loss failure [13]. A larger, prospective randomised controlled study comparing endoscopic stenting with laparoscopic washout in the management of staple-line leaks is required.

## 6 Conclusion

A staple-line leak in the post-operative period remains a severe complication. Therefore, utmost importance must be placed on the prevention of staple-line leaks. It is unfortunate that despite the best preventive measures instituted, the number of leaks encountered will increase due to the popularity of this procedure. The management of staple-line leaks remains very challenging, as there is limited systematic evidence-based literature. Therefore, we are presenting our experience to contribute to the existing literature. A larger, prospective randomised controlled study is needed to demonstrate the optimal management of this complication. It is likely that with an appropriate, tailored approach to each patient, this complication can be safely managed.

## Consent

Written informed consent was obtained from patient and patient's next of kin for publication of this case series and accompanying images. A copy of the written consents is available for review by the Editor-in-Chief of this journal on request.

## Ethical approval

Ethical approval has been exempted by our institution's ethics committee (The National University of Malaysia's Ethics Committee) as this publication is a case report, provided that patients/patient's next-of-kin have given their informed written consent for the publication of this case report.

## Sources of funding

No source of funding

## Author contribution

Study concepts: Nik Ritza Kosai, Reynu Rajan, Study design: Nik Ritza Kosai, Reynu Rajan.

Data acquisition: Loo Guo Hou, Quality control of data and algorithms: Nik Ritza Kosai, Reynu Rajan, Data analysis and interpretation: Loo Guo Hou, Statistical analysis: Not applicable.

Manuscript preparation: Loo Guo Hou.

Manuscript editing: Loo Guo Hou.

Manuscript review: Nik Ritza Kosai, Reynu Rajan.

## Conflicts of interest

No conflict of interests.

## Registration of research studies

Registered with National Medical Research Register (NMRR) Malaysia with NMRR ID number: NMRR-19-1073-48311 (UIN).

Registered with the Thai Clinical Trial Registry with TCTR identification number is TCTR20190606009.

## Guarantor

Loo Guo Hou.

## Provenance and peer review

Not commissioned, externally peer reviewed.
